# Addressing Anemia in High‐Altitude Populations: Global Impact, Prevalence, Challenges, and Potential Solutions

**DOI:** 10.1002/ajh.27761

**Published:** 2025-07-02

**Authors:** Ayoub Boulares, Nicola Luigi Bragazzi, Gustavo F. Gonzales, Paul Robach, Benoit Champigneulle, Julien V. Brugniaux, Emeric Stauffer, Elie Nader, Stéphane Doutreleau, Philippe Connes, Samuel Verges, Aurélien Pichon

**Affiliations:** ^1^ Laboratory Mobility, Aging & Exercise‐ER20296 (MOVE) Faculty of Sport Sciences‐STAPS, University of Poitiers Poitiers France; ^2^ Department of Mathematics and Statistics Laboratory for Industrial and Applied Mathematics (LIAM), York University Toronto Ontario Canada; ^3^ Human Nutrition Unit (HNU), Department of Food and Drugs Medical School, University of Parma Parma Italy; ^4^ Facultad de Ciencias de la Salud Universidad San Ignacio de Loyola Lima Peru; ^5^ National School for Mountain Sports, Site of the National School for Skiing and Mountaineering (ENSA) Chamonix France; ^6^ University Grenoble Alpes, Inserm, CHU Grenoble Alpes, HP2 Grenoble France; ^7^ Department of Anesthesia and Critical Care CHU Grenoble Alpes Grenoble France; ^8^ Laboratoire Interuniversitaire de Biologie de la Motricité (LIBM) EA7424, Team ‘Vascular Biology and Red Blood Cell’ Université Claude Bernard Lyon 1, Université de Lyon Lyon France; ^9^ Laboratoire D'excellence du Globule Rouge (Labex GR‐Ex), PRES Sorbonne Paris France; ^10^ Exploration Fonctionnelle Respiratoire, Médecine du Sport et de L'activité Physique Hospices Civils de Lyon, Hôpital Croix Rousse Lyon France

**Keywords:** anemia, correction formula, diagnostic criteria, high‐altitude

## Abstract

Anemia, a global health challenge affecting a quarter of the global population, results from diverse causes such as nutritional deficiencies, chronic diseases, and genetic factors. It disproportionately impacts women of reproductive age and children, leading to significant morbidity and mortality. While high‐altitude populations face unique diagnostic challenges due to natural hemoglobin increases, the current World Health Organization cutoffs often overestimate anemia in these regions. Altitude corrections significantly alter prevalence rates, particularly in South American children, leading to misdiagnosis. Proposed solutions include population‐specific thresholds and iron status markers like serum hepcidin, though economic constraints and limited test availability remain challenges. Tailored strategies informed by genetic research highlight adaptations in Tibetan and Ethiopian highlanders, demonstrating the need for region‐specific approaches. Socioeconomic factors exacerbate anemia in high‐altitude areas. Addressing anemia requires updated diagnostic criteria, personalized strategies, and increased awareness to ensure accurate assessments and interventions in diverse populations, especially those residing at high altitudes.

## Graphical abstract

Key challenges in diagnosing anemia in high‐altitude populations include variability in hemoglobin (Hb) concentration, associated pathological conditions, altitude‐induced plasma volume changes, and environmental factors such as contamination and nutrition. Future directions aim to improve diagnostic accuracy through altitude‐ and region‐specific Hb reference values, novel anemia biomarkers adapted to high‐altitude contexts, and personalized management strategies.

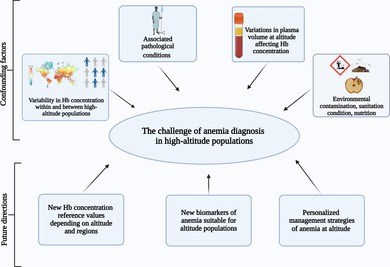

## 1 Introduction

Anemia is a major global health challenge affecting an estimated quarter of the world's population, imposing a substantial burden in terms of morbidity, mortality, and socioeconomic costs [1]. Although iron deficiency remains the most frequently cited cause, the etiological landscape of anemia is remarkably diverse. It includes deficiencies of other micronutrients (e.g., vitamin B12 and folate), hemoglobinopathies (e.g., sickle cell disease and thalassemia), and anemia of chronic disease [1, 2]. Women of reproductive age and young children are especially vulnerable, with iron deficiency anemia contributing to adverse pregnancy outcomes, impaired cognitive development, and heightened susceptibility to infection [3]. Accurate diagnosis and effective treatment are therefore pivotal to controlling this significant public health issue.

Diagnosing anemia, however, is far from straightforward. Hemoglobin (Hb) cutoffs established by the World Health Organization (WHO) provide widely accepted thresholds for identifying anemia in the general population, yet these reference values were largely developed and validated in low‐altitude and high‐income settings. Such generalized classifications often fail to capture the complexities of diverse global populations, particularly individuals living in high‐altitude regions (> 2500 m). Due to the reduced partial pressure of oxygen at high altitude, an elevated Hb concentration is a normal physiological adaptation to maintain adequate tissue oxygenation. When altitude‐dependent Hb correction cutoffs are applied in these environments, they can inadvertently inflate the prevalence of anemia, labeling many physiologically normal individuals as anemic.

Recent WHO guidelines acknowledge the need for altitude corrections, but growing evidence indicates that these one‐size‐fits‐all adjustments may still be inadequate [4]. Emerging data from Tibetan, Ethiopian, and Andean populations suggest that ethnic, genetic, and lifestyle differences further modulate Hb levels [5, 6, 7]. Similarly, coexisting factors such as smoking, alcohol consumption, chronic infections, and nutritional deficiencies introduce an additional layer of complexity, making it challenging to interpret Hb levels accurately in high‐altitude communities. Overdiagnosis not only wastes valuable health resources but may also prompt unwarranted interventions (e.g., iron supplementation in individuals who are not truly iron‐deficient), while underdiagnosis delays necessary treatments and potentially increases the risk of complications.

Against this backdrop, a nuanced approach to defining, diagnosing, and managing anemia in high‐altitude populations is urgently needed. Such an approach must incorporate both the physiological adaptations that raise Hb and the multifactorial etiologies that lower it. This paper systematically examines the global significance and burden of anemia, explores the diagnostic challenges posed by high‐altitude residence, and evaluates potential solutions, from revised Hb thresholds and region‐specific biomarkers to broader public health strategies.

We hypothesize that the universal application of Hb cutoff values derived from sea‐level populations, even when adjusted for altitude according to current international guidelines [5], systematically misclassifies anemia status in high‐altitude populations. These altitude‐adjusted thresholds may overestimate anemia prevalence and misclassify iron status due to population‐specific physiological and genetic adaptations. This review aims to demonstrate that integrating data from diverse geographic regions and ethnic groups can inform more accurate and context‐specific diagnostic strategies, ultimately improve anemia management and reduce the risks of over‐ or under‐treatment in high‐altitude settings.

## 2 The Global Burden and Diverse Etiology of Anemia

Anemia is a prevalent global health issue characterized by a reduction in the number of circulating red blood cells or a lower‐than‐normal Hb concentration, irrespective of the underlying cause such as red blood cell enzyme deficiency, red blood cell membrane disorders, hemoglobinopathies, or kidney disease, resulting in a diminished oxygen‐carrying capacity of the blood [8, 9]. Clinically, anemia is conventionally determined when Hb levels fall below the established normal threshold for age, sex, and physiological status [10, 11]. Anemia can be subdivided into acute anemia, typically resulting from sudden blood loss, or chronic anemia, which develops over time and is often associated with underlying health conditions such as nutritional deficiencies or chronic diseases. Chronic anemia is accompanied by significant adverse health outcomes, increased morbidity and mortality, and imposes substantial health and economic burdens worldwide [1, 12].

In 2021, the global prevalence of anemia, encompassing both chronic and acute forms, was 24.3%, a decrease from 28.2% in 1990 [3]. Nevertheless, the total number of individuals affected by anemia has increased due to population growth, rising from 1.50 billion in 1990 to 1.92 billion in 2021 [1]. In 2021, it accounted for 52.0 million years lived with disability (YLDs), which translates to 5.7% of all YLDs globally, making it one of the leading causes of disability worldwide [1].

Some specific populations and demographics are dramatically affected by chronic anemia. Women of reproductive age (15–49 years) face a high burden, with a prevalence of 31.2%, compared with an age‐matched rate of 17.5% in males [3]. This condition in pregnancy is associated with higher rates of preterm labor, postpartum hemorrhage, low birth weight, short gestation, stillbirth, and infections in both the mother and the child [13]. In children, anemia can impair cognitive and motor development and increase susceptibility to infections, particularly in tropical regions where severe infections such as malaria elevate the risk of childhood mortality [14]. Children under 5 years of age show a particularly high prevalence of 41.4% in an analysis conducted in 37 locations worldwide [1], including regions such as sub‐Saharan Africa, South Asia, and tropical Latin America, with a global prevalence of anemia in children aged 6–59 months estimated at 39.8% [15].

This high prevalence among children and pregnant women is further complicated by the occurrence of two specific forms of anemia that have been observed exclusively in these groups: “physiological anemia” and “pathological anemia.” In pregnant women, “physiological anemia” is caused by a hemodilution process, leading to Hb values below the threshold defined by the WHO, although this condition is considered normal. Fetal hemoglobin (HbF) is the main form of Hb during fetal development and is gradually replaced by adult hemoglobin (HbA) after birth [16]. By around 6 months of age, HbF levels typically decrease to less than 1% in normal populations, with most Hb being HbA (approximately 96%) and HbA2 making up 2.5%–3.5% [17]. Therefore, hemolysis associated with elevated HbF levels is not characteristic of healthy children beyond infancy. The original claim about hemolysis due to elevated HbF in children under 5 has been adjusted to align with these facts. This normal process results in Hb values falling below the threshold defined by the WHO, classifying it as anemia. This postnatal adjustment, described by Joseph Barcroft in 1932 as “Everest in utero,” is a normal physiological response rather than a sign of disease [18]. During this period, neonates possess high iron stores, almost double (75 mg/kg) that of adults, which adequately support erythropoiesis in early life [19]. Thus, dietary iron primarily serves to replace small daily iron losses and support growth needs. In most cases, this modest requirement is sufficiently met through breastfeeding.

In contrast, pathological anemia refers to reductions in Hb concentration due to underlying pathological processes and typically requires clinical evaluation and treatment. The causes of pathological anemia are diverse, ranging from conditions that induce blood loss, shorten red blood cell lifespan, or hinder Hb synthesis and red blood cell production. These include chronic inflammation, which disrupts iron metabolism through elevated hepcidin levels and impairs iron absorption—rendering oral iron supplementation less effective. Other etiologies include chronic kidney disease (which reduces erythropoietin production), gastrointestinal disorders (that impair nutrient absorption), malaria (which accelerates hemolysis), hemoglobinopathies such as sickle cell disease and thalassemia, red blood cell membrane disorders, red blood cell channelopathies, red blood cell enzyme deficiencies, and infections with neglected tropical diseases such as schistosomiasis and hookworm [20]. These diverse causes reflect the multifactorial nature of pathological anemia, which varies according to age, physiological status, and regional disease burden.

Adults suffering from anemia often experience symptoms like weakness, fatigue, difficulty concentrating, and reduced productivity in daily activities and work. Among older adults, anemia is known as a risk factor for hospitalization, poor surgical outcomes, and increased all‐cause mortality [1]. This has been particularly observed when moderate to severe anemia is present [21]. Due to these varied causes, pathological anemia prevalence significantly varies by geography: the highest rates can be found in regions such as western sub‐Saharan Africa (47.4%) and South Asia (43.0%), where conditions like malaria and hemoglobinopathies, such as sickle cell disease in sub‐Saharan Africa, and thalassemia syndromes in South Asia, are common. These conditions shorten the lifespan of red blood cells, contributing to the higher prevalence of anemia. In South America, anemia presents a variable burden: in countries like Colombia, El Salvador, Costa Rica, Nicaragua, Ecuador, Mexico, Peru, Honduras, and Argentina, anemia was classified as a mild public health problem, with prevalence rates ranging from 7.6% to 18.7%. In Guatemala, Brazil, the Dominican Republic, and Bolivia, anemia was considered a moderate public health issue, with prevalence between 21.4% and 38.3%. Panama and Haiti reported the highest prevalence rates at 40.0% and 45.5%, respectively, classifying anemia as a severe public health concern in these countries [22]. In contrast, regions like Australasia and Western Europe have much lower prevalence rates, around 5.7% and 6.0%, respectively [1].

Persistent high anemia burden, particularly among women of reproductive age and young children, emphasizes the urgent need for a renewed focus on accurately measuring its prevalence and underlying causes [23]. Since these groups include individuals with “physiological anemia” that does not require intervention, it is necessary to develop methods to distinguish “physiological anemia” from “pathological anemia” or “true anemia.”

## 3 The Challenges in Estimating the Global Burden of Anemia

Estimating the burden imposed by anemia is not a trivial task. The difficulties in reliably quantifying its consequences stem from complex challenges related to its diagnosis [23]. As previously mentioned, anemia is multifactorial and can result from various causes. These can often be hard to differentiate, making accurate diagnosis challenging, especially in developing countries. Also, multiple types of anemia can occur simultaneously, often presenting with similar symptoms, making it challenging to distinguish and accurately attribute the specific causes of anemia on a global scale. Achieving this requires more comprehensive, high‐quality data to understand and quantify the contributions of different underlying causes across diverse populations [24].

Over‐attribution to iron deficiency anemia, the most common type of anemia, is an important issue, often due to its status as a default diagnosis in regions with limited diagnostic resources and its high prevalence as a primary etiology in many populations [25]. Analyses often rely on iron deficiency, while only about half of anemia cases globally are due to it [26], leading to misdiagnosis and misclassification. Therefore, assessing anemia should involve testing various biomarkers (e.g., ferritin and retinol) with different sensitivities and specificities. This “biomarker complexity” is further heightened and compounded by interactions with factors like inflammation, nutrient deficiencies, and pregnancy, especially in developing countries, where multiple types of anemia can coexist.

Limitations of global standards for anemia diagnosis and outdated definitions represent other aspects of the challenge of estimating the burden of anemia. The commonly used thresholds for defining anemia are largely arbitrary. These thresholds, such as those recommended by the WHO, were initially based on Hb distributions observed in limited populations, predominantly from Western countries (Europe, the United States, and Canada) in the 1960s, and were not derived from direct associations with health outcomes. While revising these thresholds might seem straightforward, accurately defining anemia remains highly complex. This complexity arises from recognized differences in biological, geographical, and geopolitical factors, compounded by challenges related to measurement methods, test validity, and accessibility [27].

Current Hb concentration cutoffs established by the WHO for anemia diagnosis (13.0 g/dL for men aged 15 years and older, 11.0 g/dL for children, and 12.0 g/dL for nonpregnant women) may not reflect functional outcomes or current clinical practice [4, 28]. Also, they do not appear to be linked with physiological, health, or other relevant outcomes [28]. A cross‐sectional study based on 79 950 Hb observations from 30 global nutrition surveys conducted between 2005 and 2016 across 25 countries [28] revealed that WHO cutoffs are higher than the fifth percentile in most countries, except the United States [28]. This pattern was observed in 13 445 children aged 6–59 months and 25 880 nonpregnant women aged 15–49 years. Elevated levels of the soluble transferrin receptor (sTfR), a biomarker, were used to assess increased demand for iron due to red blood cell production, which can indicate iron deficiency anemia rather than other anemia types. All this has led to incomplete epidemiological definitions: anemia cutoffs should consider intersectional factors such as age, sex/gender, smoking, pregnancy, and underlying health status.

In terms of regional variation, the Biomarkers Reflecting Inflammation and Nutritional Determinants of Anemia (BRINDA) project [29] found significant variation in Hb levels among healthy women of reproductive age across different countries, suggesting that the current WHO cutoff of 11.0 g/dL may not be universally applicable. This is particularly relevant in developing countries where factors such as higher rates of infectious diseases, nutritional deficiencies, and environmental stressors (e.g., high altitude) can impact Hb levels independently of anemia. As such, addressing anemia requires a nuanced approach tailored to regional and physiological variability, particularly in high‐altitude settings. Additionally, the updated 10.5 g/dL Hb cutoff for children aged 6–23 months may still overlook the natural postnatal decline in Hb [4], which reflects normal development rather than pathology.

Besides the previously mentioned challenges, altitude represents a further intersectional factor that should be accounted for. However, it remains controversial how to quantify the rate of anemia at high altitude [30].

## 4 The Challenges in Estimating the Burden of Anemia at High Altitude

### 4.1 Physiological and Hematological Responses to High‐Altitude Hypoxia

Exposure to high‐altitude hypobaric hypoxia triggers a series of coordinated physiological and hematological adaptations aimed at preserving oxygen delivery to tissues. The decline in arterial oxygen partial pressure (PaO_2_) triggers chemoreceptor‐mediated hyperventilation, increasing alveolar oxygen partial pressure but also inducing respiratory alkalosis due to excessive CO_2_ elimination. This alkalosis initially dampens the ventilatory response but is gradually counteracted by renal bicarbonate excretion, allowing sustained hyperventilation over time. Concurrently, sympathetic nervous system activation elevates heart rate and cardiac output, temporarily enhancing oxygen delivery to tissues [31].

Another acute response to altitude is a reduction in plasma volume, which leads to a significant increase in hematocrit and Hb concentration, alongside a decrease in total blood volume. This plasma volume contraction, driven by complex fluid regulation mechanisms, contributes to the partial normalization of arterial oxygen content within the first few days of exposure. Plasma volume generally remains reduced throughout the duration of altitude exposure [32]. In parallel, elevated circulating erythropoietin (EPO) stimulates erythropoiesis in the bone marrow, promoting increased red blood cell production and a resulting rise in Hb concentration, thereby enhancing further the blood's oxygen‐carrying capacity [33] and contributing to the normalization of cardiac output. Therefore, lowlanders acclimatizing at high altitudes as well as permanent high‐altitude residents, generally exhibit elevated Hb concentrations [32]. These physiological adaptations are driven by a cascade of molecular events initiated by oxygen‐sensing pathways, particularly the stabilization of hypoxia‐inducible factors (HIF‐1α and HIF‐2α), which upregulate the transcription of EPO in the kidneys and liver. Under sustained hypoxic conditions, additional layers of physiological and genetic adaptations emerge, contributing to long‐term acclimatization in high‐altitude populations [34].

### 4.2 Population‐Specific Adaptations to High‐Altitude Environments

The physiological and genetic adaptations observed among long‐term high‐altitude residents offer compelling insights into how human populations have evolved to cope with chronic hypobaric hypoxia. Across the globe, such populations have developed distinct physiological and genetic adaptations shaped by evolutionary pressures and environmental contexts. For instance, Andean highlanders typically show significantly elevated Hb levels as their principal adaptive mechanism, supported by genetic variations in cardiovascular and erythropoietic regulatory genes, such as *NOS2A, EDNRA*, and *BRINP3*, enhancing blood oxygen‐carrying capacity [35]. More recently, evidence from Aymara highlanders has identified population‐enriched alternative transcripts of *NFKB1*, which may contribute to high‐altitude adaptation through modulation of inflammatory pathways and hypoxia responses [36]. Conversely, Tibetan highlanders, who have resided above 4000 m for approximately 25 000 years, display Hb concentrations similar to sea‐level populations despite chronic hypoxia. This unique phenotype results from genetic adaptations in the *EPAS1* and *EGLN1* polymorphisms—key regulators of the HIF pathway—combined with elevated ventilation rates and higher nitric oxide production, promoting vasodilation and increased blood flow without excessive red blood cell production [37]. Beall's study further highlights that Tibetans exhibit significantly higher resting ventilation and hypoxic ventilatory response (HVR) compared to Andeans, facilitating greater oxygen intake [7]. In contrast, Andeans display lower ventilation and HVR but rely more heavily on increased Hb concentrations, illustrating divergent evolutionary strategies to manage chronic hypoxia [7].

Ethiopian highlanders also present distinct adaptive patterns, characterized by near‐normal Hb and hematocrit values despite living at altitudes above 3500 m [38, 39]. Their adaptive phenotype involves genetic variations in oxygen‐sensing and response pathways, including genes such as *CBARA1*, *VAV3, ARNT2*, and *THRB*, which enhance oxygen utilization without increased erythropoiesis [40]. Notably, most of these genetic adaptations have not been identified in previous studies involving high‐altitude Tibetan or Andean populations [40], despite similar altitudes of residence. Furthermore, physiological factors like the reduction in plasma volume seen in Andean populations contribute to their higher Hb levels [41, 42, 43]. These pronounced inter‐population heterogeneities in response to hypobaric hypoxia highlight the limitations of employing standardized altitude‐correction formulas derived predominantly from a few populations to determine abnormal Hb levels. Consequently, accurately diagnosing anemia at high altitudes necessitates the development of population‐specific criteria that account for regional physiological variability and genetic heterogeneity.

### 4.3 Historical Evolution of Hb Adjustment Approaches for High‐Altitude Anemia Diagnosis

Over the decades, multiple formulas have been proposed to standardize Hb adjustments for altitude, yet no single approach has achieved universal acceptance (Table 1). This evolution traces back to the late 1950s and 1960s, when the WHO established universal anemia thresholds largely derived from sea‐level data. At that time, high‐altitude residents were underrepresented, even though their elevated Hb due to hypoxia was well documented. By 1989, WHO and other public health agencies formally recognized the need for altitude corrections, advocating an increase in the anemia cutoffs to account for higher baseline Hb. Through the 1990s and early 2000s, guidelines (e.g., WHO/UNICEF and CDC) introduced specific tables for altitude and smoking adjustment, typically adding a fixed increment to the anemia threshold depending on elevation. However, these methods were often based on limited empirical data, chiefly pre‐1985 studies of Andean men and U.S. children, raising concerns about their global applicability. In the years since, various data‐driven and physiologically modeled formulas have aimed to refine or replace these early corrections, but genetic, ethnic, and regional differences remain insufficiently captured. Most recently, in March 2024, the WHO released updated guidelines on Hb cutoffs for defining anemia in individuals and populations, introducing a revised equation for altitude adjustments starting at 500 m above sea level [4]. This revision represents the culmination of extensive, multiyear reviews integrating diverse data sources, notably impacting anemia definitions for children aged 6–23 months, now established at Hb < 10.5 g/dL. The newly recommended equation modestly increases anemia prevalence at moderate elevations (between 500 and 3000 m) while reducing it at altitudes above 3500 m—an attempt to address earlier formulas' tendency to over‐ or under‐correct in certain regions. However, a concern with this guideline is that arterial oxygen saturation and oxygen transport do not vary significantly between 0 and 1500 m, and no symptoms are observed at these altitudes. According to the new guidelines, individuals living between 500 and 999 m should have their measured Hb value adjusted downward by 0.4 g/dL [51].

**TABLE 1:** Altitude adjustment formulas and values (1945–2024).

Year	Source	Formula	Categorization
1945	Hurtado et al. [44]	Adjustment refers to the value either added to the Hb threshold used to define anemia or subtracted from an individual's measured hemoglobin level.	Data‐driven
1968	WHO	No specific formula provided. Recognized the need to adjust for altitude but did not provide quantitative guidance.	Not applicable
1989	CDC [45]	Hb_adjustment (g/L) = [(−0.032 × (altitude × 0.0032808) + 0.022 × (altitude × 0.0032808)^2^) × 10]	Data‐driven
1994	Dirren et al. [46]	Hb (g/L) = 3.44 × exp.(0.000633 × altitude) + 116.9	Data‐driven
2001	WHO/CDC [47]	Adjustment (g/dL) = −0.032 × *A* +0.022 × *A* ^2^ Where *A* = altitude (m) × 0.0032808	Data‐driven
2011	WHO [9]	Adjustments provided directly in a table; no formula specified.	Data‐driven
2018	Silvana Ocas‐Cordova et al. [48]	Hb (g/dL) = 1.19e−07 × altitude^2^ +0.0001123 × altitude+11.2964	Data‐driven
2019	Sharma et al. [49]	Hb adjustment (g/L) = 0.0048108 × altitude+0.0000004 × altitude^2^	Data‐driven
2020	Silubonde et al. [50]	Cutoff threshold of 12.35 g/dL for 1700 m	Data‐driven
2023	Mairbäurl et al. [42]	ΔHb (g/dL) = 0.1 × [−0.32 × 0.0033 × altitude+0.22 × (0.0033 × altitude)^2^]	Data‐driven
2024	Proposed WHO [4] Guidelines	Adjustment (g/L) = 0.0056384 × altitude+0.0000003 × altitude^2^	Data‐driven

Abbreviations: CDC, Centers for Disease Control and Prevention; Hb, hemoglobin; WHO, World Health Organization.

### 4.4 Current Limitations and Population‐Specific Challenges in Hb Adjustment

#### 4.4.1 Regional and Demographic Variability in Hb Responses

Approximately 81.6 million people, or about 1.1% of the global population, reside permanently at elevations above 2500 m (8200 ft) [52]. This altitude places them at potential risk for polycythemia [53]. The WHO recommends adjusting the Hb cutoff points to define anemia at higher elevations, acknowledging that Hb levels naturally increase with altitude. For instance, a study conducted on Peruvian children found that Hb levels remain relatively constant from sea level up to 999 m [48]. Above this altitude, Hb levels begin to rise, increasing from an average of 11.32 g/dL at 1000 m to approximately 14.54 g/dL at 4000 m, which reflects a physiological adaptation to the reduced oxygen availability at higher altitudes. WHO's approach to correcting the Hb distribution curve for altitude increases the proportion of subjects classified with anemia while reducing the proportion with Hb > 14.5 g/dL or excessive erythrocytosis (i.e., Hb levels above 21 g/dL in men and 19 g/dL in women [54]), which may diminish potential adverse health implications of high Hb levels [55].

On the other hand, the increase in Hb with altitude is neither universal nor linear [30, 48, 56], as observed in the Himalayas, where Tibetans, who have lived in highlands for more than 25 000 years, exhibit lower Hb concentrations than people of the Han ethnicity, who have resided there for no more than 70 years [57]. A comparative quantitative study [58], employing a Bayesian meta‐analytical approach with meta‐regressions, revealed that the Andean population exhibits the highest increase in Hb, with a rate of 1 g/dL per 1000 m of altitude, while other regions show a smaller increase, averaging 0.6 g/dL per 1000 m. Recent studies further highlight significant regional variability in Hb responses to altitude, with South Americans and East Africans such as the Amhara and Oromo demonstrating the highest Δ[Hb]/km, while South/South‐East Asians and Middle Eastern populations exhibit smaller, and in some cases, even decreases in children at similar altitudes [42].

Despite these continuous revisions, the WHO recommendations for correcting Hb cutoffs based on altitude, which rely primarily on data mostly available from Western countries (Caucasian) and South American highlands, lack global generalizability [57]. A recent scoping review [57] synthesized 14 studies and found that most published investigations used small sample sizes sampled in a non‐randomized fashion and used an analytical cross‐sectional design.

Furthermore, Hb levels are generally lower in older adults compared to younger populations, adding another layer of complexity. In populations such as infants, children, pregnant women, and adults (males and females), the prevalence of anemia using altitude‐corrected Hb is found to be significantly higher—three to five times—compared to using markers of iron status alone [42, 59]. Recent studies indicate that demographic differences further influence Hb responses to altitude. For instance, children exhibit Δ[Hb]/km values up to 25% lower than adults, and pregnant women show reduced increases due to plasma volume expansion [42, 60]. Additionally, when correcting Hb levels for altitude, the frequency of erythrocytosis appears to decrease, further complicating the assessment. For example, Ocas‐Córdova et al. [48] examined the Hb levels in 2 028 701 Peruvian children aged 6–59 months living at various altitudes, from 2012 to mid‐2017. Using the WHO Hb threshold of 11 g/dL without altitude correction, the prevalence of anemia was about 35% for children below 1000 m and 4.5% for those above 4000 m. After altitude correction, the prevalence was found to be similar below 1000 m, but a staggering 66% above 4000 m. Regarding polycythemia, using a threshold of Hb levels greater than 14.5 g/dL without altitude correction would increase its prevalence from 1.2% below 1000 m to 39.4% above 4000 m. After altitude correction, the prevalence was found to decrease with altitude.

Another study conducted in Peru corroborated these findings. Gonzales et al. [55] evaluated diverse populations, including adults, infants, and children, to determine the validity of the correction proposed by the WHO. The findings showed significant changes in anemia prevalence with Hb correction. In infants from Puno (3800 m), the prevalence of anemia rose dramatically from 11.3% to 94.7% after correction. Similarly, in children under 60 months in the Arequipa region (2500 m), the prevalence increased from 16.1% to 50% post‐correction. In adults diagnosed with anemia based on corrected Hb values, ferritin levels were approximately 76.7% higher than those diagnosed using uncorrected Hb thresholds, highlighting the potential misclassification of individuals with sufficient iron stores. Similarly, body iron content and total iron reserves were 9.2% greater in individuals classified as anemic using corrected Hb values, further reinforcing the issue of misclassification [55].

Importantly, individual‐level factors such as pregnancy, smoking, and alcohol consumption may exert differential effects on Hb concentrations depending on altitude. For instance, a large Peruvian cohort study (35 000 pregnancies) reported that both low (< 9 g/dL) and high (≥ 14.5 g/dL) maternal Hb levels were associated with adverse outcomes such as stillbirth, preterm birth, and small‐for‐gestational‐age infants at both low and high altitudes [61], suggesting that elevated Hb at altitude in pregnancy is not always protective and may even be detrimental [62].

In contrast, the interaction between smoking and altitude has been less extensively studied. However, the findings by Ramirez et al. [63] indicate that smoking at 3000 m leads to a further elevation in Hb and hematocrit levels beyond the already elevated values found in high‐altitude dwellers, reflecting likely additive effects of cigarette‐induced and altitude‐induced hypoxia. These hematological changes occurred without significant differences in erythropoietin levels or pulmonary function between smokers and non‐smokers [63]. Smoking alone has been shown to elevate Hb concentrations even at sea level, potentially leading to underdiagnosis of anemia in smokers [64]. Current guidelines already recommend adjusting Hb thresholds for smoking and altitude independently [4]. This compounded effect may further obscure the accurate diagnosis of anemia, leading to the misclassification of individuals who do not exhibit iron deficiency but present with elevated Hb due to these external factors.

Regarding alcohol consumption, a large cross‐sectional analysis from the Ethiopian Demographic and Health Survey found a significant inverse association between alcohol intake and anemia in nonpregnant reproductive‐age women. Women who consumed alcohol, regardless of frequency, had higher Hb concentrations and significantly lower odds of anemia compared to non‐drinkers, even after adjusting for confounders, including altitude and smoking [65]. Furthermore, upward migrants often exhibit distinct physiological responses compared to Indigenous high‐altitude populations, including differences in erythropoietic drive and oxygen utilization, which could lead to misclassification when applying uniform Hb correction formulas across populations with different levels of adaptation.

These imprecise and unreliable measures hamper the effectiveness of public health interventions. Indeed, despite state programs aiming to control anemia through iron supplementation, these efforts often prove ineffective for high‐altitude populations, making the occurrence of iron deficiency at high altitude a contentious issue. This could be explained, at least partially, by the different iron metabolism of high‐altitude residents regulated by key molecules like EPO, hepcidin, and erythroferrone, which could cause lower responses to iron supplementation [66]. Exposure to high altitude increases the body's demand for iron, and hepcidin levels tend to decrease in response to hypoxia, allowing for increased iron absorption and release from stores, thereby enhancing erythropoiesis [66, 67].

#### 4.4.2 Consequences of Misclassification and the Need for Better Markers

**FIGURE 1:**
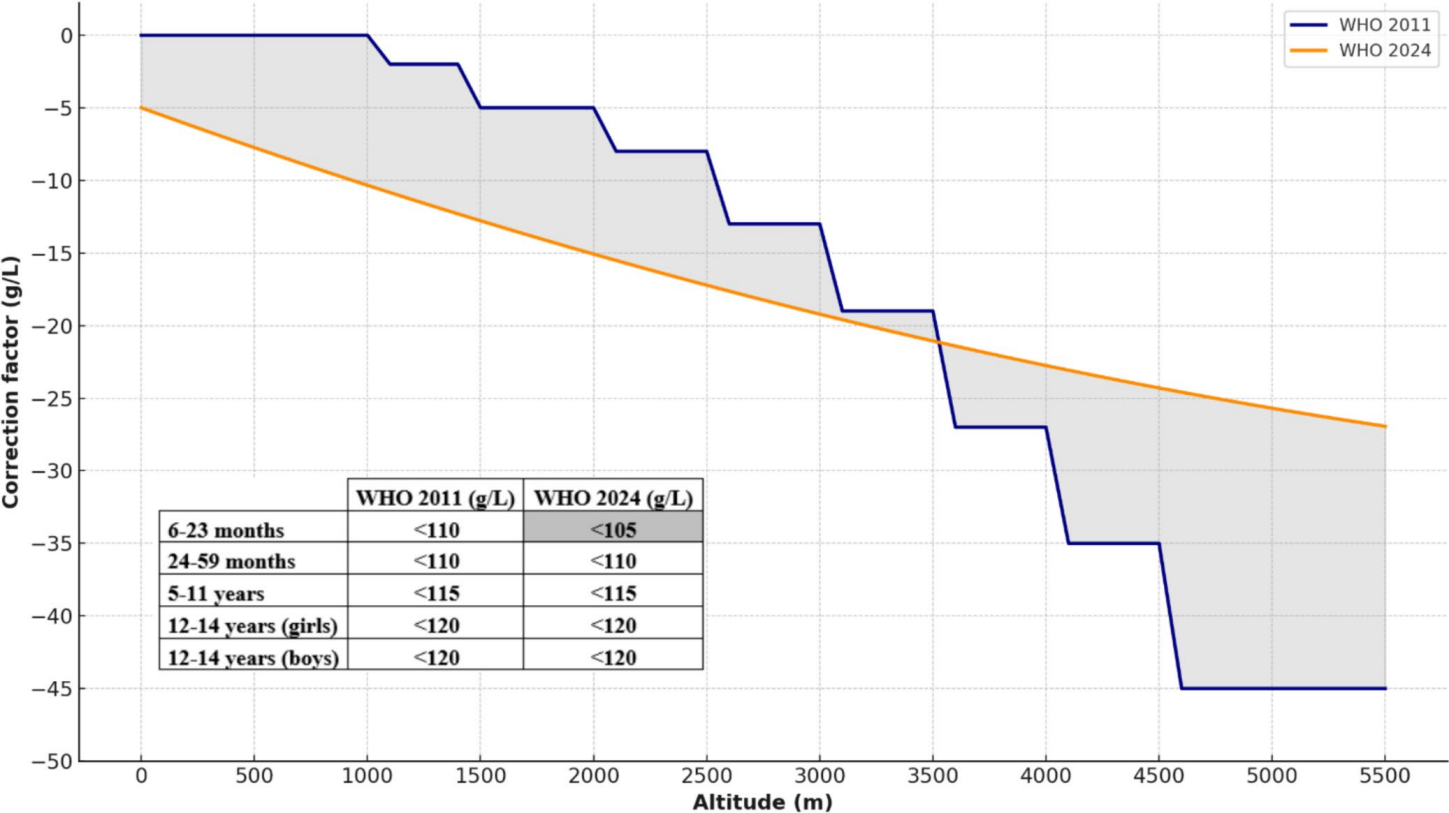
Comparison of altitude‐dependent hemoglobin correction factors: WHO 2011 vs. WHO 2024. The embedded table summarizes anemia thresholds at sea level for children and adolescents as defined by each guideline. [Color figure can be viewed at wileyonlinelibrary.com]

Clinical outcomes in the study by Gonzales et al. [55] illustrate the risk of overestimating anemia prevalence due to inappropriate Hb correction formulas. In pregnant women, those classified as anemic based on altitude‐corrected thresholds showed fewer adverse outcomes, such as preterm birth and stillbirth, than those classified as anemic without altitude correction. This finding suggests that current correction methods may overestimate the anemia burden and inappropriately label healthy individuals anemic [13]. Furthermore, applying these corrections significantly reduced the number of children classified as having normal nutritional status without anemia, reinforcing the concern that altitude‐adjusted thresholds may distort both clinical and nutritional assessments. Figure 1 presents an implementation example comparing the WHO 2011 and WHO 2024 altitude‐dependent Hb adjustments for children from 6 to 23 months. This comparison underscores how the choice of correction method can significantly influence anemia classification, particularly at high altitude.

While most research on altitude correction has focused predominantly on Andean populations, recent studies reveal that similar, and even more severe, misclassification issues affect other high‐altitude populations with distinct physiological adaptations. For instance, Sarna et al. [68] reported that Tibetan highlanders residing at approximately 3932 m exhibit mean Hb concentrations of 15.6 ± 1.5 g/dL in men and 14.2 ± 1.1 g/dL in women, values roughly 1 g/dL lower than the current WHO altitude‐adjusted thresholds. When the WHO adjustments (originally derived from Andean data) were applied to this Tibetan sample, the apparent prevalence of anemia soared to 77.8% in men and 86.5% in women, despite being healthy, non‐smoking, and iron‐sufficient individuals.

Similarly, evidence from East Africa further illustrates this variability. In a study conducted by Sarna et al. [69] on Ethiopian highlanders, two closely related ethnic groups—the Amhara residing at 3700 m and the Oromo at 4000 m—were examined. Without altitude adjustments, using only age‐ and sex‐specific thresholds, none of the individuals were classified as anemic. However, with the WHO's age–sex–altitude adjusted thresholds, the apparent prevalence of anemia increased dramatically: to 28.3% among Amhara men and 48.5% among Amhara women, compared with 9.1% and 14.3% among Oromo men and women, respectively.

These studies collectively illustrate a critical limitation in current altitude correction approaches: formulas developed primarily from Andean data fail to account for the diverse evolutionary strategies employed by different high‐altitude populations. This cross‐population evidence strengthens the case for developing population‐specific diagnostic criteria rather than relying on universal altitude correction formulas. The consequences of misclassification extend beyond statistical concerns to impact clinical decision‐making and public health resource allocation, potentially leading to unnecessary interventions in healthy individuals while failing to identify those truly in need of treatment. Future research must focus more extensively on Tibetan and Ethiopian populations, who remain underrepresented in the literature despite their unique adaptations to high‐altitude environments.

Compounding these challenges, most large‐scale epidemiological studies in the Andean rely solely on Hb measurements to diagnose anemia, overlooking critical iron biomarkers (e.g., ferritin and serum transferrin receptor) and inflammation markers (e.g., CRP and IL‐6). For instance, a large‐scale cohort of over two million Peruvian children (6–59 months) across all regions relied on Hb values (via finger‐prick HemoCue) and altitude data from health surveys, with no biochemical iron or inflammatory biomarkers collected [48]. Similarly, a 2020 study of more than 11 000 Peruvian children, drawing on the National Demographic and Health Survey (ENDES), accounted for factors like sanitation and household fuel use but measured only Hb, with no ferritin or CRP data recorded [70]. Such omissions can inflate anemia rates by misconstruing physiologically elevated Hb concentrations as a deficiency or, conversely, mask genuine iron‐deficiency anemia under hypoxia‐driven polycythemia. Only in recent years have some studies begun measuring iron and inflammation biomarkers, revealing that incorporating these indicators provides a more accurate distinction between true anemia and high‐altitude adaptation [71, 72].

If adjusting the Hb cutoff points for high‐altitude to determine iron deficiency is inadequate and may not accurately reflect the true anemia burden in these populations, what can be the solutions?

### 4.5 Alternative Strategies: Local Thresholds and Biomarkers

#### 4.5.1 Population‐Specific Thresholds and Iron Status Markers

Not using correction for altitude would decrease the prevalence of anemia by 5%–83% [57], with the greatest drop observed at the highest altitudes. There are also proposals for new or adapted/modified formulas. For instance, Ocas‐Córdova et al. [48] proposed a new threshold for anemia, defined as Hb levels below the local mean minus two standard deviations, which resulted in a prevalence of 15% below 1000 m and 5% above 4000 m. Defining polycythemia by using the new threshold of Hb levels, its prevalence decreased at high altitudes [73]. Similarly, Accinelli et al. [70], in their analysis of the factors contributing to anemia and its prevalence in Peruvian children aged 6–35 months in data drawn from the ENDES, used the 5th percentile as a cutoff. Applying this correction, anemia prevalence was computed at 7.3% in 2016 and 2017, which was significantly lower than the WHO's reported prevalence of around 43.7%. In the same vein, Cohen and Haas [74] developed an exponential Hb‐altitude model using data from non‐anemic women living between 1000 and 4800 m in Bolivia. When applied to pregnant women in La Paz (3.600 m) and El Alto (4000 m), this model provided higher and more realistic estimates of iron deficiency anemia compared to other widely used correction methods, underscoring the need for altitude‐ and population‐specific adjustments.

Several studies have shown that the WHO recommendations for Hb correction at altitude may result in misclassification of anemia, leading to an overestimation of anemia prevalence [42, 48, 49, 55]. While the guidelines aim to account for physiological changes at altitude, they lack adaptability for population‐specific variations. Taken together, these findings underscore the need for systematic comparisons of methods (sex/gender‐specific, age‐specific, region‐specific, corrected for clinical, laboratory, or sociodemographic parameters) to evaluate which approach is the most meaningful and relevant.

Another way to avoid misclassifications could be to look at iron status markers, such as serum hepcidin levels, which are essential for regulating iron homeostasis and availability. In the study by Gonzales et al. [55], hepcidin levels in adults at high altitude were similar to those at low altitude, showing no significant iron deficiency despite anemia classification based on corrected Hb levels. Likewise, infants from Puno with normal uncorrected Hb levels (11–14.5 g/dL) had higher serum ferritin levels than those classified with mild or moderate anemia. While ferritin levels reflect actual iron stores and remained unaffected by Hb correction, the correction misclassified many infants with normal iron stores as anemic. However, it is important to note that ferritin is also an acute‐phase protein and can be elevated in response to inflammation, potentially masking depleted iron stores [75].

However, it is important to recognize that hepcidin expression is influenced by multiple factors beyond iron status. Acute exposure to altitude has been shown to suppress hepcidin levels [76], enhancing iron availability during increased erythropoietic demand. Inflammatory states, conversely, can upregulate hepcidin, contributing to anemia of inflammation by limiting iron absorption and mobilization [77]. Moreover, ancestral background may also modulate hepcidin responses to hypoxia, as evidenced by Lundgrin et al. [78], who observed distinctive plasma hepcidin profiles among Ethiopian highlanders with chronic hypoxic exposure. Taken together, these findings underscore the need for multi‐marker approaches that consider not only Hb levels but also inflammatory and genetic factors to improve the accuracy of anemia diagnosis in high‐altitude populations.

Altogether, these data seem to suggest that iron deficiency might not be as prevalent at high altitude as previously thought and that the pathogenesis of anemia at high altitude could be related to other factors [70, 79]. In a study conducted in Peru [70], children from households using solid fuels or without access to safe drinking water had higher anemia rates. The link between solid fuel use and anemia may be mediated by exposure to indoor air pollution, which induces systemic inflammation and impairs erythropoiesis. However, this association may also reflect broader socioeconomic determinants, as households relying on solid fuels often face multiple forms of deprivation, chronic malnutrition, limited access to healthcare, and poor sanitation, all of which contribute to lower Hb levels and a higher prevalence of anemia.

Given the substantial inter‐population and individual variability in physiological responses to high altitude, there is a pressing need to develop region‐specific diagnostic criteria and management strategies that reflect these unique adaptations. Moreover, this variability also underscores the importance of advancing personalized approaches to assess and manage high‐altitude illnesses more effectively.

#### 4.5.2 Emerging Diagnostic Tools, Biomarkers, and Genomic Insights

Large‐scale genome sequencing studies in underrepresented populations can advance our knowledge and help define the genetic footprints of high‐altitude adaptation [80, 81, 82]. For instance, a study conducted on 1001 indigenous Tibetans across various regions of the Qinghai–Tibetan Plateau in China [80] successfully identified 34.7 million variants, with 36% being novel. More specifically, 4320 high‐confidence adaptive variants were identified, affecting 192 genes, and four new genes were found to have strong signals of selection related to cardiopulmonary functions. However, while genomics and multiomics approaches hold promise for advancing our understanding of anemia and other conditions, their implementation may not be cost‐effective in low‐ and middle‐income countries (LMICs).

Recent technological advancements, however, have created unprecedented opportunities for genomic research in resource‐constrained settings. The continuous reduction in sequencing costs—from approximately $100 million for the initial human genome sequencing in 2000 to less than $1000 currently—has substantially enhanced accessibility [83]. Concurrently, innovative implementation strategies are emerging, including the integration of next‐generation sequencing into clinical diagnostics for rare and congenital disorders and the development of locally maintained bioinformatics pipelines that facilitate independent analysis and interpretation of genomic data [84]. These developments underscore a growing recognition of the need to establish biobanking infrastructure in LMICs to capture population‐specific genetic diversity and support longitudinal investigations, including the study of variants associated with high‐altitude adaptation [85]. This is particularly important given that approximately 78% of participants in genome‐wide association studies have been of European ancestry, while only 10% are of Asian descent, 2% are African, 1% are Hispanic, and all other ethnicities represent less than 1%, leaving the vast majority of human genetic diversity largely unexplored [86].

Leveraging these resources, targeted genomic approaches present a practical, cost‐effective alternative to whole‐genome sequencing. In particular, specialized genetic panels derived from local biobank data and validated through indigenous bioinformatics capabilities allow the identification of clinically relevant, population‐specific biomarkers that would otherwise remain undetected in European‐centric genomic databases.

Additionally, point‐of‐care (POC) assays represent another promising approach for LMICs. These field‐deployable diagnostic tools can simultaneously measure multiple biomarkers relevant to anemia assessment, including Hb concentration, iron status indicators, and inflammation markers, without the need for sophisticated laboratory infrastructure. Recent advances in microfluidics and biosensor technologies have enabled the development of affordable, easy‐to‐use devices capable of delivering rapid, comprehensive hematological profiles in remote settings. A recent systematic review documented the successful use of point‐of‐care Hb tests (POC(Hb)Ts) in clinical settings across 16 LMICs, underscoring their feasibility and growing relevance in resource‐limited healthcare systems [87].

In this context, incorporating the assessment of iron status, through biomarkers such as ferritin and sTfR, alongside Hb concentration monitoring, may constitute a practical and effective strategy to enhance the detection of iron‐deficiency anemia in high‐altitude populations. Furthermore, the inclusion of CRP in this panel could facilitate the accurate interpretation of ferritin levels by accounting for inflammation‐related variations, thereby improving the estimation of body iron stores. While hepcidin remains a promising research marker due to its central role in iron regulation, its high cost and sensitivity to numerous biological confounders limit its clinical applicability [88]. Similarly, erythroferrone remains experimental and expensive. Prioritizing cost‐effective, validated biomarkers in POC formats could facilitate more accurate, context‐specific anemia diagnosis, especially in remote or high‐altitude environments with constrained resources.

Emerging research has identified potential new biomarkers that could enhance our understanding of physiological adaptations to high‐altitude environments. Specifically, *N*‐acylethanolamides (NAEs)—endogenous fatty acid derivatives known to bind to peroxisome proliferator‐activated receptor (PPAR) subunits α and γ—have shown promise in this regard. NAEs also modulate the endocannabinoid system, a signaling pathway activated during physiological stress, making them relevant candidates for biomarker exploration. In a metabolomic study, plasma levels of four NAEs—palmitoylethanolamide (PEA), oleoylethanolamide (OEA), stearoylethanolamide, and linoleoylethanolamide—were measured in populations native to high‐altitude Puno (3830 m, Peru) and low‐altitude Lima (150 m, Peru). The study found that all NAEs were significantly elevated in the high‐altitude population. Notably, individuals with the highest NAE levels tended to exhibit both the highest Hb concentrations and the lowest pulse oxygen saturation. Although no direct association was found between NAE levels and chronic mountain sickness scores, these findings suggest that PEA and OEA may serve as exploratory biomarkers for monitoring physiological regulation and long‐term adaptation to high‐altitude exposure [89]. However, their utility in diagnosing anemia remains speculative at this stage and warrants further investigation.

These findings indicate polygenic and pleiotropic effects of adaptation. In this context, the diagnosis of anemia should not rely solely on Hb concentration, as it may overlook variations in plasma volume that significantly affect oxygen transport [90, 91]. Incorporating further novel biomarkers, such as total Hb mass measurement, offers a more accurate evaluation of anemia in high‐altitude populations. More recently, the calculation of arterial oxygen content (CaO_2_), based on the measurements of Hb and arterial oxygen saturation, has been demonstrated to be superior as a marker to determine the oxygen transport capacity in low‐altitude and high‐altitude populations than the use of Hb levels alone [71].

While the classical definition of anemia at low altitudes is well‐established, characterized by specific Hb thresholds and associated symptoms such as fatigue, increased heart rate, and reduced exercise capacity that directly impact the quality of life and survival [92], the clinical relevance of Hb variations at high altitudes remains insufficiently understood. Although differences in Hb levels may reflect the variability of physiological adaptations to high altitude, there is often a discrepancy between laboratory measurements and clinical outcomes. Elevated Hb levels do not necessarily correlate with measurable outcomes such as fatigue, cardiovascular strain, or overall morbidity. Furthermore, the potential influence of Hb variations on survival rates and susceptibility to altitude‐related illnesses has yet to be fully explored. Bridging this knowledge gap requires targeted research correlating Hb levels with measurable clinical outcomes. Establishing symptom‐based Hb thresholds tailored to high‐altitude environments could, in theory, enable more accurate interventions and help avoid unnecessary treatments such as iron supplementation, which may carry risks like iron overload. While such thresholds remain largely unexplored in high‐altitude settings, this approach has shown value in other clinical contexts, such as chronic kidney disease or heart failure, where anemia management is increasingly guided by symptom burden and functional outcomes rather than fixed Hb cutoffs alone [93]. This highlights a potential avenue for future research in high‐altitude populations.

**FIGURE 2:**
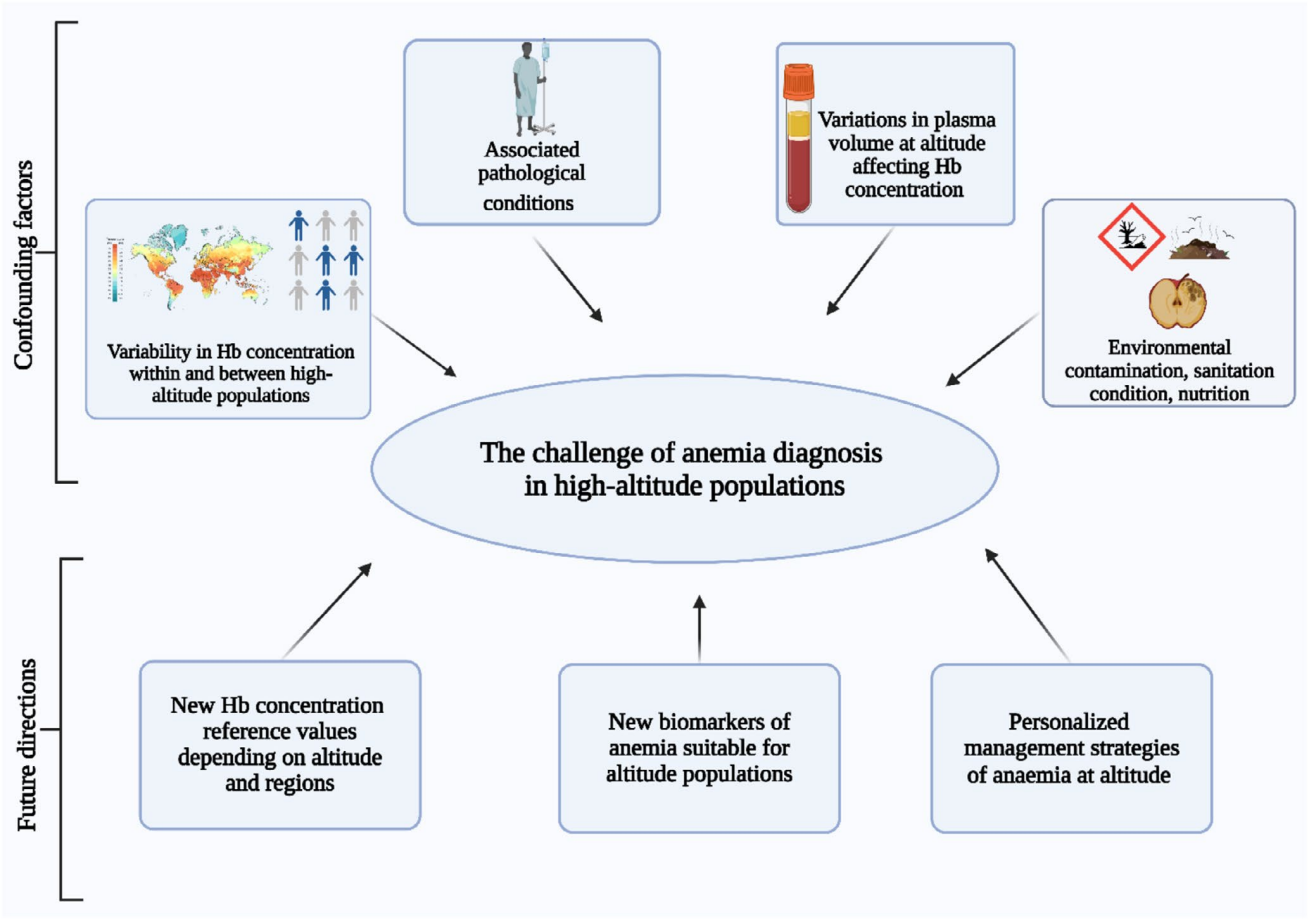
Challenges and future directions in anemia diagnosis for high‐altitude populations (created with BioRender.com, accessed on April 11, 2025). [Color figure can be viewed at wileyonlinelibrary.com]

Finally, raising public and clinical awareness about the distinct presentation and management of high‐altitude illnesses compared to lowlanders is crucial for ensuring effective prevention and treatment strategies. Figure 2 summarizes the key challenges in diagnosing anemia in high‐altitude populations and outlines future directions.

## 5 Conclusion

Anemia remains a significant global health challenge, particularly in high‐altitude populations where physiological adaptations complicate standard diagnostic practices. Current WHO Hb cutoff points may lead to misclassification, either under‐ or overestimating anemia in high‐altitude regions. To address this, a refined approach is needed, incorporating revised diagnostic thresholds tailored to population‐specific adaptations, alongside personalized clinical assessments and emerging biomarkers. Additionally, integrating genetic, socioeconomic, and environmental factors will enhance diagnostic precision and treatment efficacy. Implementing this comprehensive framework would facilitate greater diagnostic precision, enable more targeted therapeutic interventions, and enhance health outcomes for the populations most impacted.

## Author Contribu‑tions

A.B., N.L.B., and A.P. conceived the study, developed the research framework, and wrote the first draft of the manuscript. G.F.G., P.R., B.C., J.V.B., E.S., E.N., S.D., P.C., and S.V. reviewed the manuscript, provided constructive feedback, and validated the final version. All authors approved the submission of the final manuscript.

## Ethics Statement

The authors have nothing to report.

## Consent

The authors have nothing to report.

## Conflicts of Interest

The authors declare no conflicts of interest.

## Data Availability

As this is a narrative review, no original data were generated or analyzed during this study. All data discussed in this review are derived from previously published studies.
